# T cell metabolism in kidney immune homeostasis

**DOI:** 10.3389/fimmu.2024.1498808

**Published:** 2024-12-16

**Authors:** Zikang Liu, Binbin Dai, Jiwen Bao, Yangbin Pan

**Affiliations:** ^1^ Department of Nephrology, Shanghai Pudong Hospital, Fudan University Pudong Medical Center, Shanghai, China; ^2^ Center for Medical Research and Innovation, Shanghai Pudong Hospital, Fudan University Pudong Medical Center, Shanghai, China

**Keywords:** T cell, cellular metabolism, kidney disease, microenvironment, immune homeostasis

## Abstract

Kidney immune homeostasis is intricately linked to T cells. Inappropriate differentiation, activation, and effector functions of T cells lead to a spectrum of kidney disease. While executing immune functions, T cells undergo a series of metabolic rewiring to meet the rapid energy demand. The key enzymes and metabolites involved in T cell metabolism metabolically and epigenetically modulate T cells' differentiation, activation, and effector functions, thereby being capable of modulating kidney immune homeostasis. In this review, we first summarize the latest advancements in T cell immunometabolism. Second, we outline the alterations in the renal microenvironment under certain kidney disease conditions. Ultimately, we highlight the metabolic modulation of T cells within kidney immune homeostasis, which may shed light on new strategies for treating kidney disease.

## Highlights

- The metabolism of T cells mediates their functions through both metabolic and epigenetic mechanisms.
- T cells play a crucial role in maintaining immune homeostasis in the kidney, and their abnormal involvement contributes to the onset and progression of kidney diseases.
- Kidney pathology alters the microenvironment, modulating T cell metabolism, which in turn exacerbates the disease.
- Modulating T cell metabolism offers a novel therapeutic strategy for targeting certain kidney diseases.

## 1 Introduction

Immune homeostasis is a prerequisite for normal human growth and development (1). Perturbation of immune homeostasis can lead to the development of a broad spectrum of diseases, such as infection, chronic inflammation, metabolic disease, and cancer (2–6). T cells are pivotal in maintaining immune homeostasis, as they exert multiple functions, including pathogen resistance, immune regulation, immune memory, and tumor surveillance. To execute these functions, naïve T cells originate in the bone marrow and mature in the thymus, but proliferation and differentiation occur in secondary lymphoid tissues such as lymph nodes. Therefore, it is widely established that the presented antigen, costimulatory signals, and cytokines are three major signals for T cell activation.

In the process of exerting immune function, T cell subsets undergo flexible metabolic reprogramming to meet bioenergetic demands. Specifically, T cells are classified into multiple subsets based on distinct surface markers and corresponding functions, among which CD4^+^ T cells and CD8^+^ T cells are predominantly investigated. In a quiescent state, naive CD4^+^ and CD8^+^ T cells mainly rely on oxidative phosphorylation (OXPHOS) for energy supply (7, 8). However, once activated, the corresponding T cell subset undergoes metabolic reprogramming, which entails a rewiring from mitochondrial processes to glycolysis and glutaminolysis (9–11). In addition, lipid metabolism regulates energy or biomass production, membrane structure, and signal transduction in T cells, thus serving as a key meditator of T cell function (12). The functional integrity of T cells is tightly coupled to metabolic reprogramming. Remarkably, based on the critical positioning of metabolism within T cells, the nutrients, derived from energy metabolism, are posited as 4th signal for T cell immunity, in addition to the three aforementioned signals (13).

To varying extents, the perturbation of immune homeostasis, governed by the T cell, exists within either autoimmune kidney diseases mediated by autoantibodies directly or indirect immune kidney diseases evidenced by immune cell infiltration (14, 15). Direct immune-mediated kidney diseases involve ones such as membranous nephropathy (MN) and anti-glomerular basement membrane(anti-GBM) glomerulonephritis, whereas indirect immune-mediated kidney diseases encompass Anti-neutrophil cytoplasmic antibody-associated vasculitis (AAVs), IgA nephropathy (IgAN), lupus nephritis (LN) (14), as well as diabetic kidney disease (DKD) and angiotensin II-induced kidney injury (15, 16). Pioneering studies have unveiled that modulating T cells' metabolic pathways during their differentiation, proliferation, effector function, and exhaustion can significantly alter their function and longevity. These metabolic interventions provide therapeutic potential, either by enhancing anti-tumor immune efficacy or by restoring immunological self-tolerance and homeostasis (17, 18). In this review, we mainly summarize the role of T cell metabolism in renal immunity. Besides, we give a brief overview of the latest advancements in T cell immunometabolism and emphasize the kidney microenvironment in certain disease contexts, which may orchestrate the fluctuation of T cell metabolism.

## 2 An overview of cellular metabolism in T cell

### 2.1 Glucose metabolism

Quiescent T cells primarily rely on fatty acid oxidation (FAO) and OXPHOS to sustain cell survival. Upon activation, the metabolic pathway of the T cell undergoes the transition from FAO to glycolysis to partially fuel the rapid metabolic demand for cell division, differentiation, and effector function, therefore the glycolytic pathways are regarded as major symbols for T cell activation (7, 8, 12, 19). A broad spectrum of glucose transporters (GLUT), key enzymes, and metabolites are involved in glucose metabolism, which meticulously modulates the biological activities of T cells.

T cells uptake extracellular glucose with the involvement of GLUT, predominantly GLUT1, GLUT2, and GLUT3 (20, 21). GLUT enhances T cell proliferation, differentiation, and function by fueling glycolysis. To elaborate, upon TCR stimulation, high expression of GLUT1 on the surface of T cells is tightly coupled to enhanced acquisition of effector phenotype, amplified proliferation, and a reversed CD4/CD8 ratio (increased CD8^+^ and decreased CD4^+^ T cells) (22). In the presence of GLUT1 activation inhibitor, the differentiation of naive CD4^+^ T cells into T helper 17 (Th17) and Th1 subsets decreased, thus exerting an immunosuppressive phenotype (23). Also, GLUT1 enhances the proliferation of CD4^+^ T cells and cytokine generation of Th1 and Th17, but not Treg and CD8^+^ T cells (24). Intervening GLUT1-mediated glucose uptake may partly attenuate auto-immune response (24). Impaired anti-tumor capacity is coupled with decreased GLUT1 and HK2 in CD8^+^ T cells (25), which, in an alternative perspective, corroborates the importance of glycolysis in maintaining T cell functionality. In addition, GLUT1 could coordinate with GLUT2, and facilitate glucose transport for CD8^+^ T cells in a context-dependent manner. Specifically, GLUT2 has a high affinity for glucose and oxygen. In a poorly oxygenated microenvironment, hypoxia-inducible factor-1α (HIF-1α) indirectly downregulates the expression of GLUT2 by enhancing the production of Gal-9, which in turn suppresses stomatin leading to the destabilization of GLUT2 at the cell membrane. In this context, undiminished GLUT1 compensates the glucose uptake for further effector functionality (21). However, in type 2 diabetes (T2D) individuals, hyperglycemia impairs memory CD8^+^ T cells by insulin-derived GLUT1 upregulation and enhanced glucose uptake (26). Additionally, GLUT not only unilaterally provides the energy necessary for cellular survival activities, but enables unexpected epigenetic remodeling. For instance, GLUT3-dependent glucose consumption facilitates Th17-derived cytokine production (IL-2, IL-17A, IL-17F, and GM-CSF) by histone acetylation at corresponding gene loci (27).

Pyruvate Kinase M2 (PKM2), a key enzyme in the glycolysis pathway, has aroused widespread interest in the metabolic field for its tight linkage with the "Warburg effect". Intriguingly, T cells also possess this characteristic. By reinforcing glycolysis, PKM2 can promote the differentiation of Th1 and Th17 cells, thereby inducing autoimmunity (28). From an epigenetic perspective, PKM2 translocates into the nucleus where it integrates with STAT1/3/4, thus modulating Th1 and Th17 differentiation (29, 30). The translocation, however, can be impeded by diet-derived vitamin B5, which is catabolized into coenzyme A (CoA), thereby binding PKM2 and inhibiting Th17 differentiation (31). Inflammatory hepatic CXCR3^+^ Th17 cells (ihTh17) in non-alcoholic fatty liver disease exhibit enhanced glycolysis, mediated by PKM2, leading to the transition towards inflammatory phenotype (32). In lupus, the PKM2 agonist known as TEPP-46 dampens follicular helper T cell (Tfh) differentiation by modulating the transcription factor BCL6 and inhibiting glycolysis, validating the vital role that PKM2 plays in Tfh differentiation (33). Similarly, TEPP-46 can hinder the differentiation of both Th1 and Th17 cells, thus attenuating autoimmunity (30). In addition, TEPP-46 can inhibit the differentiation of Th17, as well as Treg, independent of PKM2 and its metabolic function, via hindering Smad2/Smad3 signaling pathway activated by TGF-β (34). Of note, the aforementioned conclusions present a certain contradiction: based on the properties of TEPP-46 in activating PKM2, glycolysis should be theoretically enhanced; however, the T cell subsets exhibit impaired glycolysis. One plausible explanation is that dimeric PKM2 is capable of translocating to the nucleus and increasing gene transcription, such as HIF-1α, mTORC1, and Myc, and the utilization of TEPP-46 reverses the modulation. On the one hand, TEPP-46 enhances the activity of PKM2. On the other hand, TEPP-46 impairs the transcription of partial genes, which are indispensable for glycolysis. In T cell subsets, the application of TEPP-46 shifts the balance toward inhibiting glycolysis.

Lactate, a key metabolite derived from glycolysis, is recognized for its pronounced regulatory capacity in T cell metabolism and epigenetics. Due to the characteristic that tumors mainly utilize aerobic glycolysis for energy supply, human tumor cells produce up to 40 times more lactate than normal cells, thus furnishing a lactate-rich microenvironment. Despite that lactate can be theoretically reused for oxidation in mitochondrial, the data from tumor research revealed that lactate impedes the CD8^+^ T cells mediated tumor surveillance (35, 36). In the CD4^+^ T cell subsets, the capacity of producing IL-17A is blunted in lactate-treated Th17 cells, while the Foxp3 expression is enhanced, suggesting an ongoing lineage transition from Th17 to Treg cells (37). However, in chronic inflammatory disease, the accumulated lactate is not only a metabolite within glycolysis but also acts as a signaling molecule, which is conveyed into CD4^+^ T cells through SLC5A12-mediated uptake, leading to increased IL-17 production via nuclear PKM2/STAT3 and the vulnerability of tissue-resident (38). One plausible explanation for the two conflicting phenotypes is that one highlights lactate's epigenetic modulation, whereas the other regards lactate as a signaling molecule, with each grounded in a distinct biological context employing different experimental methodologies. Additionally, in the lactate-rich context, the suppression of the Treg cells promotes the initiation and progression of tumors (39). Further, in the highly glycolytic microenvironments, lactate impedes Treg cell function by upregulating PD-1 expression on its surface (40).

**Figure 1:**
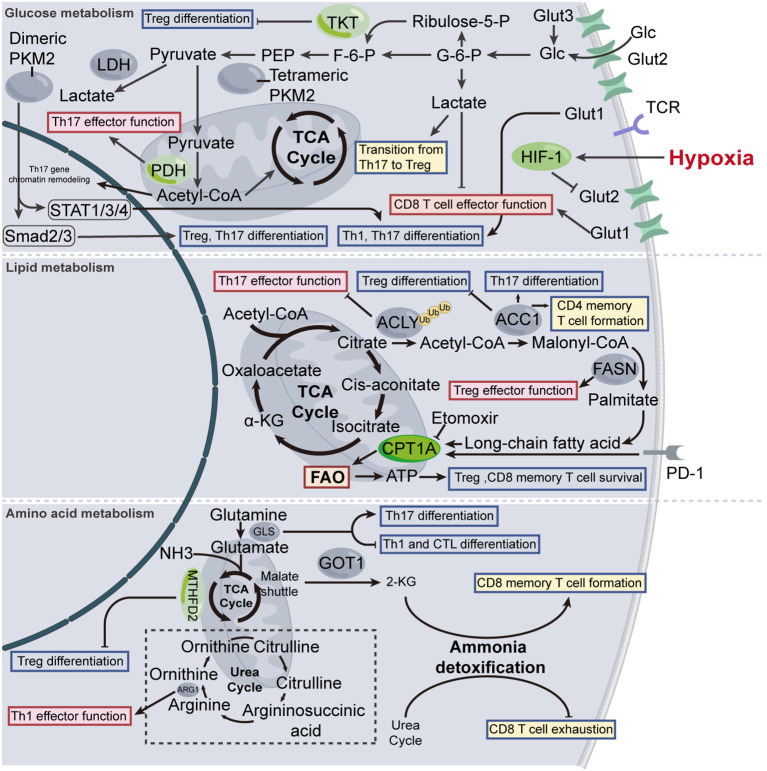
Metabolic modulation of T cell. The normal functioning of biological activities necessitates the support of energy metabolism. Fluctuation in T cell metabolic levels modulates T cell activation, differentiation, and effector functions to a certain extent. TKT, transketolase; LDH, lactate dehydrogenase; PEP, phosphoenolpyruvate; F-6-P, fructose-6-phosphate; G-6-P, glucose-6-phosphate; Glc, glucose; GLUT, glucose transporters; PKM2, pyruvate kinase isozymes M2; ACLY, ATP citrate lyase; ACC1, acetyl-CoA carboxylase 1; FASN, fatty acid synthase; CPT1A, carnitine palmitoyl-transferase 1A; FAO, fatty acid oxidation; GLS, glutaminase; GOT1, glutamic-oxaloacetic transaminase 1; ARG1, arginase1; 2-KG, 2-ketoglutarate.

Pyruvate dehydrogenase (PDH) is the key enzyme controlling the pyruvate flux that engages in the tricarboxylic acid (TCA) cycle. A loss-of-function study revealed that the metabolic rewiring towards glutaminolysis and glycolysis, and the lipid uptake following PDH deficiency leads to impaired Th17 effector function (41). In this context, the enhanced glutaminolysis incited by PDH deficiency does not appear to replenish the impairment of Th17 cell function, suggesting the promotive effect of glutamate metabolism on Th17 cell function still needs to be reflected in the citrate pool within the TCA cycle. The pentose phosphate pathway (PPP) is divided into two stages: the first stage is the oxidative phase, and the second stage is the non-oxidative phase, which is also referred to as non-oxidative PPP. The first stage provides the cell with ribose-5-phosphate and NADPH, as required, while the second stage highlights the reusability that the excess pentose phosphates could be redirected back into the glycolytic pathway. Specific deletion of transketolase (TKT), the only enzyme involved in non-oxidative PPP that catalyzes two reversible reactions, impairs Treg differentiation via reducing glycolysis and exacerbating oxidative stress, consequently leading to fatal autoimmune damage in mice (42) (**Figure 1**).

### 2.2 Lipid metabolism

Lipid plays a pivotal role in T cell fate decision. First, it serves as an essential component of cellular membranes. Second, lipids uptake, *de novo* synthesis, and hydrolysis are closely linked with intracellular homeostasis of lipids, thereby modulation T cell subset differentiation and function. The synthesis and catabolism of lipids hold equivalent importance in T cells, as they not only provide energy for T cell biological activities but also regulate T cell responses. During the process, A series of key enzymes involved in the catalytic reactions, including ATP citrate lyase (ACLY), acetyl-CoA carboxylase 1 (ACC1), fatty acid synthase (FASN) and carnitine palmitoyl-transferase 1A (CPT1A) etc.

ACLY orchestrates the interconnection between glucose metabolism and *de nove* lipogenesis by catalyzing the formation of cytosolic acetyl coenzyme A (AcCoA) from mitochondria-released citrate (43). Treg cell differentiation is accompanied by impaired enzymatic activity of ACLY, while increased expression of ACLY is coupled with improved Th17 cell responses in either metabolomic or epigenetic manner (27, 44–46). ACC1 catalyzes the first rate-limiting step in the synthesis of palmitic acid. Inhibition of ACC1 results in the suppression of Th17 cell formation and concurrently promotes the augmentation of Treg cell and memory CD4^+^ T cell development (45, 47). Conversely, the anti-tumor capacity of CD8^+^ T cells can be enhanced by inhibiting ACC1, mainly because of the restricted lipid utilization (48). FASN is a critical multifunctional enzyme complex responsible for catalyzing the biosynthesis of long-chain fatty acids. FASN-mediated *de novo* fatty-acid synthesis improves the function of Treg cells. Conversely, deficiency of FASN in Treg cells alleviates tumor growth (49).

CPT1A, the rate-limiting enzyme of FAO, orchestrates the migration of AcCoA from the cytoplasm into the mitochondrial matrix. In general, most CD4^+^ T cell subsets, including Th1, Th2, and Th17 cells, metabolically rely on aerobic glycolysis, whereas Treg cells, as well as CD8^+^ memory T cells, are mainly reliant on FAO (50–52). In this context, the therapeutic approach of targeting CPT1A to enhance cellular FAO, thereby improving T cell function, has emerged. For instance, butyrate, a specific type of short-chain fatty acid (SCFA), could improve FAO in Treg cells, which in turn enhances its differentiation, thereby attenuating autoimmune response (53). PD-1 facilitates the metabolic rewiring of activated T cells from glycolysis to FAO by upregulating CPT1A, thus exerting the immunosuppressive capacity (54). In certain contexts, albeit specific knockout of CPT1A in T cells, the CPT1A inhibitor, etomoxir still exhibits the capacity of modulating Treg cell, as well as CD8^+^ memory T cell differentiation, suggesting the existence of alternative bioenergetic fueling mechanism beyond FAO (55).

Long-chain fatty acid (LCFA) plays a pivotal role in sustaining metabolic fitness in CD8^+^ T cells. In the LC-FAs family, linoleic acids positively mediate the abundance and biosynthesis of mitochondria in CD8^+^ T cells via formatting ER-mitochondria contacts, thereby potentiating immune response, longevity, and memory (56). In addition to the effect on CD8^+^ T cells, LCFAs are capable of enhancing differentiation and proliferation of Th1 and Th17 cells (57). In contrast, SCFAs promote Treg differentiation and function, thereby playing a role in suppressing autoimmune response (57).

Sterols are important components of cell membranes, where they play critical roles in maintaining membrane structure and fluidity. liver-X receptor (LXR) is a key regulator of sterol transportation and synthesis, the form of which, known as LXRα and LXRβ, are identified as expressed in T cells (58, 59). LXR target gene encodes Srebp-1, thereby suppressing Th17 differentiation. In contrast, genetic deletion of LXR promotes Th17 polarization in mouse CD4^+^ T cells. Pharmacological activation of LXR (GW3965, TO901317) suppresses Th17 cell differentiation in autoimmune diseases (60, 61), however, reduces myeloid-derived suppressor cell abundance, thus boosting cytotoxic T lymphocyte (CTL) responses and enhancing its anti-tumor capacity (62) (**Figure 1**).

### 2.3 Amino acid metabolism

Ammonia is conveyed in the bloodstream as glutamine, which is subsequently hydrolyzed into glutamate in the context of glutaminase (GLS). *In vivo*, GLS deficiency leads to impaired differentiation of Th17 cells, however, boosts the differentiation of Th1 and CTL cells (63). Glutamine blockade increased CD8^+^ cell immune potency, manifested as enhanced potent antitumor responses (64). For CD8^+^ T cells, excessive ammonia leads to cell death, and at different time points, CD8^+^ T cells possess distinct mechanisms for ammonia detoxification. In the memory phase, CD8^+^ T_M_ cells eschew ammonia-mediated cytotoxicity by harnessing the urea cycle and citrulline cycle (65). Later in the phase of T cell exhaustion, the malate shuttle takes the baton. The malate shuttle is widely recognized to sustain NAD^+^/NADH balance between the cytosol and mitochondria. During this process, glutamic-oxaloacetic transaminase 1 (GOT1) catalyzes the transition from 2-ketoglutarate (2-KG) and aspartate to oxaloacetate and glutamate. In a model of chronic viral infection, specific deletion of Got1 in T cells results in decreased NAD^+^/NADH ratio and excessive ammonia load in CD8^+^ exhausted T cells, thus impeding T cell longevity. However, rather than increasing NAD^+^/NADH, supplementing 2-KG restores the antiviral capacity of CD8^+^ T cells (66). These data have validated that glutamine metabolism, orchestrating energy supply and ammonia detoxification, maintains T cell functionality and longevity.

In the conversion of arginine to ornithine, Arginase (ARG)1 plays a pivotal role in its catalytic function. The impaired catalytic capacity of Arg1 leads to decreased consumption of arginine, but increased utilization of glutamine, for ornithine generation, which subsequently blunts availability of glutamine flux for TCA cycle (67). Loss of Arg1 in CD4^+^ T cells results in adaptive enhanced utilization of glutamine for ornithine generation, thereby impeding Th1 cell effector response which is translationally manifested as alleviated lung tissue injury during influenza infection (67).

Methylenetetrahydrofolate dehydrogenase 2 (MTHFD2) is a one-carbon (1C) metabolism enzyme, that is involved in modulating *de novo* purine synthesis. Due to its crucial role in the synthesis of DNA and RNA, as well as in cell repair and regeneration, MTHFD2 functions as a metabolic checkpoint in inhibiting the Treg cells differentiation and transdifferentiation of Th17 to Treg cells (68). Itaconate, a metabolite in the TCA cycle, recently shed light on the therapeutic strategy for ameliorating autoimmunity, due to its profound anti-inflammatory effect. Specifically, itaconate can inhibit glycolysis and OXPHOS, which leads to decreased levels of certain metabolites, termed S-adenosyl-L-methionine/S-adenosylhomocysteine(SAM/SAH) ratio and 2-hydroxyglutarate (2-HG). These metabolites respectively bind to the Il17a and Foxp3 loci, modulating the activity of RORγt, a transcription factor that belongs to the nuclear receptor family. Through these mechanisms, itaconate impedes Th17 differentiation while potentiating Treg differentiation (69) (**Figure 1**).

## 3 Kidney microenvironment

Cell metabolism is intricately linked to the extracellular milieu, collectively referred to as the cellular microenvironment; this plastic environment provides a venue for nutrient fueling, cellular signaling, cell-cell crosstalk, and dynamic sensing and adaptation to disease context (70–72). Similarly, whether infiltrating T cells or tissue-resident T cells, their differentiation, proliferation, and effector functions are, in part, governed by the microenvironment (73). For instance, tumor cells and Treg compete for the limited nutrient supply within the tumor microenvironment (TME), forcing Treg to make metabolic adaptations to meet the fluctuating energy demand for their survival and functionality (73). Still, in solid tumors, SENP7 senses oxidative stress in the TME and translocates from the nucleus to the cytoplasm, where it regulates PTEN deSUMOylation. This process subsequently modulates the glycolysis and OXPHOS of CD8^+^ T cells, reinforcing their antitumor functionality (74). In addition, the hepatic microenvironment in fatty liver disease is characterized by lipid deposition, oxidative stress, chronic inflammation, and fibrosis. FABP1 orchestrates FA uptake, facilitating the accumulation of T_RM_ in liver (75). Non-alcoholic steatohepatitis (NASH) impairs the mitochondrial function and glucose uptake of CD8^+^ T cells, diminishing its anti-tumor capacity. Whereas, metformin can reshape the metabolic fitness of CD8^+^ T cells (76). In the context of liver-specific metabolic regulation, invariant natural killer T (iNKT) cells exert a protective effect against acute liver injury, while the deletion of PKM2 in iNKT cells impairs their activation, thereby diminishing their protective effects on the liver (77). In the cardiac microenvironment of myocarditis, characterized predominantly by inflammatory cytokine infiltration, inhibition of PGK1 in CD4^+^ T cells can alleviate cardiac inflammation and fibrosis (78).

In the kidney, a persistent topic of discussion is how disease alters the renal microenvironment, and how these changes in the microenvironment promote pathological alterations in renal intrinsic cells (79). Despite the unresolved issues, the dynamic changes in the renal microenvironment associated with certain kidney diseases dramatically endow T cells with the capacity for cellular metabolic rewiring, thereby modifying the progression of the disease (80).

### 3.1 Lactate

Throughout the development and progression of renal diseases, the aberrant metabolic function in the kidney has been well established. Given that a broad-spectrum enzyme and metabolites are involved in the process of metabolic energy supply for the kidney, the metabolic homeostasis can be perturbed by any given step within the metabolic pathway, thus leading to kidney damage. Among this, lactate is notably highlighted for its distinct metabolic niche, as pyruvate is shunted at this point; under hypoxic conditions, it is converted to lactate, otherwise, it enters the TCA cycle. However, an exceptional scenario exists wherein pyruvate undergoes conversion to lactate for energy demand, even under conditions of sufficient oxygenation. This phenomenon is known as the Warburg effect (81).

Mounting studies have unveiled that the cellular metabolic pathway within the kidney is prone to aerobic glycolysis, a seemingly inefficient mode of energy supply with excessive lactate production (82–85). In the model of unilateral ischemia/reperfusion-induced acute kidney injury (AKI) to chronic kidney disease (CKD), increased levels of PKM2 lead to renal pericyte transdifferentiation by enhancing PKM2 nuclear translocation and lactate dehydrogenase (LDH)A and GLUT1 transcription, which in turn increase lactate production (83). In the context of DKD, elevated lactate levels in renal kidney proximal tubule and urine, may be attributable to an impaired TCA cycle, indicating that the tubulointerstitium in DKD exhibits a high lactate state. A well-substantiated study, using samples of abundant patients with CKD and 3 mouse models of CKD, demonstrated that impaired renal gluconeogenesis is capable of orchestrating the prediction of adverse renal outcomes (86). Given that gluconeogenesis is considered an effective pathway for alleviating tissue lactate load, irrespective of the diverse etiologies, patients with CKD exhibit a certain degree of renal tissue lactate accumulation. Given the heterogeneity of partial renal cells, lactate is an indispensable factor in exploring the pathogenic mechanisms of T cell metabolism in the kidney.

### 3.2 Hypoxia

Hypoxia is a pathological condition in which the scarce oxygen cannot adequately fuel the organ or the body. The kidney is a metabolically rich organ, and an adequate oxygen supply is a prerequisite for normal cellular function. The majority of blood in the kidney fuels the renal cortex, attributing to the only 10%-15% blood supply to the renal medulla. In this context, when the kidney encounters hypoxia, the renal medulla is more susceptible to damage (87). To a lesser extent, a broad spectrum of kidney diseases is associated with hypoxia, although the regulatory mechanisms, such as HIFs (88), are triggered to meet the cellular bioenergetic demands.

AKI is categorized into prerenal, intrinsic, and postrenal types depending on the etiology, with each category exhibiting different extents of renal tissue hypoxia (89). Furthermore, Hypoxia not only causes renal injury but may also be secondary to other diseases, thereby exacerbating kidney damage. For instance, loss of the rich peritubular capillary network, the prominent feature of CKD, hypoxia serves both as a consequence and cause (90). Furthermore, by impairing mitochondrial function, and triggering inflammation, oxidative stress partly contributes to tissue hypoxia under certain disease context (91). In addition, glomerular hyperfiltration and albuminuria in DKD, lead to the subsequent hypoxia in proximal tubule (PT) (92).

Based on the existing evidence, kidney injury, and the hypoxia renal microenvironment have a bidirectional causal relationship, and in the context of hypoxia, T cells may exhibit complex and precise metabolism adaption.

### 3.3 Lipid accumulation

Lipid dysregulation is a significant manifestation in renal diseases, and the following lipid accumulation in renal tissue exacerbates existing kidney damage, which is termed lipotoxicity (93, 94). Lipid dysregulation involves multiple processes, including fatty acid metabolism, cholesterol metabolism, and lipid droplet deposition, and the primary structural units affected are the renal tubular epithelial cells and podocytes (85, 94). To be specific, renal tubular epithelial cells mainly rely on FAO for energy supply, and impaired FAO renders lipids accumulated in tubulointerstitium (93). Accordingly, the patients with tubulointerstitial fibrosis exhibit lower expression of key enzymes and regulation in FAO compared to healthy individuals, and the subsequent *in vivo* and *in vitro* experiments also corroborated with this conclusion (95). The data from a large cohort have demonstrated a positive correlation between CPT1A and eGFR, which highlights the indispensable role of CPT1A for kidney survival. Further, by overexpressing the Cpt1a gene in 3 models of renal fibrosis, the study identified the therapeutic role of CPT1A, which restores FAO and significantly ameliorates renal fibrosis (96).

However, such as the vascular endothelium, mesangial cells, and podocytes, mainly rely on glycolysis and OXPHOS to fuel cellular energy demand. For this reason, the lipid accumulation within podocytes is predominately governed by the limited number of mitochondria and mitochondrial dysfunction (94). In the angiotensin II-induced podocyte injury model, both glycerol-3-phosphate and cholesterol are found to increase in glomeruli (97, 98). Furthermore, DKD involves damage to both glomeruli and tubules. Fu et al. revealed that, in DKD, junctional adhesion molecule-like protein (JAML) boosts podocyte lipid accumulation and subsequent podocyte damage via SIRT1-mediated SREBP1 signaling (99). Further study demonstrated that meteorin-like gene product alleviates mitochondrial dysfunction and lipid accumulation in renal tubules via Sirt3-AMPK signaling axis (98). ATP-binding cassette A1 (ABCA1) is a plasma membrane protein, that modulates cholesterol efflux, thus sustaining cellular cholesterol homeostasis. Loss-of-function study found that in the context of DKD, ABCA1 deficiency in glomerular endothelial cells worsens cellular cholesterol accumulation and glomerular endothelium injury (100).

Tremendous evidence has revealed that normal kidney function is attributed to lipid homeostasis. With the progression of kidney disease, the increasing accumulation of lipids within the kidney exerts lipotoxicity on kidney cells on the one hand. On the other hand, it may orchestrate metabolic reprogramming in immune cells such as T cells and macrophages.

## 4 Cellular metabolism of T cell subsets and renal immune homeostasis

The thymus is the primary site for T cell development and differentiation. During the early stage of development, the T cells exhibit expression of both CD4^+^ and CD8^+^ and are termed double-positive T cells. Subsequently, within the thymic cortex, the T cells engage with MHC molecules on the surface of thymic epithelial cells. Those T cells that bind with MHC class I molecules differentiate into CD8^+^ T cells, while those that bind with MHC class II molecules differentiate into CD4^+^ T cells (1). From an etiological perspective, T cells boost the onset of a spectrum of kidney diseases, including immune-related and non-immune related (101). Thus, in the therapeutic strategies of immune-related kidney diseases, such as lupus nephritis, membranous nephropathy, and IgA nephritis, immunosuppressants are extensively used. Given that conventional immunosuppressants are characterized by low specificity, multiple side effects, and the induction of immune tolerance, a range of monoclonal antibody therapeutics have emerged, such as rituximab, belimumab, and eculizumab (102–104). Recent studies have increasingly targeted immunometabolism, aiming to regulate immune response via cellular metabolic modulation. Among these, T cell metabolic modulation is widely discussed, and these outstanding achievements may shed light on the treatment of kidney diseases.

### 4.1 Metabolic modulation of CD4^+^ T cell subsets in kidney

Naïve CD4^+^ T cells differentiate into Th cells, including Th1, Th2, Th17, Treg, and Tfh cells, to exert regulating properties. The splenic CD4^+^ T cells undergo enhanced glycolysis and mitochondrial metabolism to meet the energy demand for aberrant autoimmune response. In the context of systemic lupus erythematosus (SLE), the administration of corresponding metabolic inhibitors, as metformin for OXPHOS and 2-Deoxy-D-glucose (2-DG) for glycolysis, reduce cytokine production of CD4^+^ T cells and ameliorate kidney damage (**Table 1**) (105). Further, through this metabolic intervention, the pre-existing immune tolerance could be amplified. In SLE123 mice (a model of lupus), altered glycosylation patterns, incited by 2-DG and metformin, enable the enriched anti-CD45RB induced-immune tolerance to be established, thereby preventing the renal deposition of auto-antibodies (106). Additionally, the utilization of glucose transporter inhibitor, termed CG-5, also enables the alleviation of aberrant autoimmune response, accompanied by increased Treg cell differentiation while diminished Th1 and Th17 cell differentiation in lupus (**Table 1**) (23). Briefly, 2-DG and CG-5 ameliorate renal injury by inhibiting glycolysis in CD4^+^ T cells, while metformin alleviates renal damage through suppression of OXPHOS.

**Table 1:** Metabolic modulation for T cell metabolism in the kidney.

Drug	T cell subsets	Target	Effect	Disease model	Ref
2-Deoxy-D-glucose	CD4^+^ T cell	Glycolysis	Cytokine production↓	Lupus	Yin et al. (105)
Metformin	CD4^+^ T cell	OXPHOS	Cytokine production↓	Lupus	Yin et al. (105)
CG-5	Th1, Th17	GLUT	Differentiation↓	Lupus	Li et al. (23)
KN93	Th17	GLUT, glycolysis	Differentiation↓	Lupus	Koga et al. (114)
FK866	CD4^+^ T cell	Glycolysis, OXPHOS	Effector function↓	Lupus	Li et al. (119)
canagliflozin	CD4^+^ T cell	Glycolysis, OXPHOS, glutaminolysis	Effector function↓, Proliferation↓	Lupus	Jenkins et al. (122)
JHU083	CD4^+^ T cell, CD8^+^ T cell	glutaminolysis	Activation, proliferation↓	AKI	Lee et al. (126)

↓, indicates a decrease or reduction.

Liver kinase B1 (LKB1) is a bioenergetic sensor that controls Treg metabolism and function. Treg-specific deletion of LKB1 leads to decreased FAO and mitochondrial dysfunction, as well as diminished intracellular ATP, decoupling from either AMPK signaling or the mTORC1-HIF-1α axis, and this metabolic reprogramming dampens Treg function, which in turn inducing autoimmune response (107, 108), as well as kidney damage (109). Similarly, in the context of insufficient optic atrophy 1 (OPA1), LKB1, as a sensor of mitochondrial perturbation, orchestrates mitochondrial membrane disruption, which hinders Th17 effector function. In line with previous studies, this process is independent of mTOR and AMPK (110).

Calcium/calmodulin-dependent protein kinase 4 (CaMK4) is a serine/threonine kinase that is found excessively activated in T cells of individuals with SLE (111). Further, a study has demonstrated that CAMK4 phosphorylates the glycolysis rate-limiting enzyme 6-phosphofructokinase, platelet type (PFKP) in Treg, increasing glycolysis while inhibiting OXPHOS. This metabolic rewiring ultimately inhibits Treg differentiation (112). CaMK4 can also regulate the Tfh-dependent transcription factor B cell lymphoma 6 (Bcl6) at the transcriptional level, thereby enhancing humoral immune-mediated autoimmune kidney damage (113). Conversely, the administration of CAMK inhibitor (KN93) impeded either glycolysis or GLUT1 in naïve T cells from MRL/lpr mice and isolated CD4^+^ T cells from patients with SLE, consolidating the metabolic modulation property of CAMK (**Table 1**) (114). Therefore, KN93 alleviates renal injury by inhibiting CaMK4, reducing downstream glycolysis, and enhancing OXPHOS.

The hypoxic kidney microenvironment typically occurs after pre-existing kidney damage, whereas renal-infiltrating CD4^+^ and CD8^+^ T cells express HIF-1, which senses hypoxia and prevents their apoptosis. In the presence of LN, specific deletion of HIF-1 in T cells decreases kidney injury (115). In a mouse model of renal ischemia-reperfusion injury (IRI), specific deletion of HIF-2α in dendritic cells relieves the suppression of CD36 expression, which in turn facilitates the hyperactivation of natural killer T cells by mobilizing the lipid uptake, ultimately exacerbating the renal injury in IRI. However, CD36 blockade by sulfo-N-succinimidyl oleate restores the kidney injury (116). In the context of obesity, lupus mice exhibit exacerbated kidney damage (117). The intriguing finding, to some extent, bridged a previous study, which demonstrated that obesity, coupled with an elevated level of CPT1A, fortified the mitochondrial E3 ubiquitin ligase known as Goliath, thereby promoting glycolysis and hyperactivation of CD4^+^ T cells (113). Hence, Goliath, as a potential therapeutic target, influences CD4^+^ T cell activation by modulating glycolysis levels.

T-cell immunoreceptor with Ig and immunoreceptor tyrosine-based inhibitory motif domain (TIGIT) is a novel immune checkpoint molecule that is conventionally seen as the "brakes" of immune function. However, the increased expressions of TIGIT in kidney T cells, due to AKI, lead to unexpected renal inflammation, as well as altered OXPHOS and mTORC1 in Th17 cells at the transcriptional level, which may in part explain the renal damage (118). A recent study has identified nicotinamide phosphoribosyltransferase (NAMPT), which facilitates the biosynthesis of nicotinamide adenine dinucleotide (NAD^+^), as a metabolic checkpoint of CD4^+^ T cells in lupus nephritis for its prominent pro-inflammatory. Mechanically, NAMPT enhanced aerobic glycolysis and mitochondrial respiration in CD4^+^ T cells, thus enhancing its capacity to produce IFNγ. Conversely, pharmacological inhibition of NAMPT by FK866 or genetic deletion of NAMPT inhibits the progression of inflammation and reduces renal damage (**Table 1**) (119). To summarize, FK866 inhibits NAMPT, suppressing glycolysis and OXPHOS in CD4^+^ T cells, thereby alleviating renal injury.

The advent of sodium-glucose co-transporter 2 (SGLT2) inhibitors shed light on the therapeutic avenue for renal disease management. Beyond their established benefits in tubular-dependent glucose lowering, diuresis, and proteinuria attenuation, recent studies have unveiled notable immunomodulatory effects exerted by SGLT2 inhibitors (120–122). Canagliflozin, an FDA-approved SGLT2 inhibitor, exerts distinct different regulatory effects on different T cell subsets. To be precise, in antitumor immunity, canagliflozin could dramatically hinder PD-L1 expression, thus enhancing CD8^+^ T cell-mediated cytotoxicity (121). However, in the CD4^+^ T cells derived from patients with SLE, the utilization of canagliflozin blunt TCR signaling, which in turn inhibits mTORC1 and Myc pathway, ultimately impeding CD4^+^ T cell function by metabolic suppression (impaired glutamine anaplerosis, glycolysis, and OXPHOS) (**Table 1**) (122). Hence, SGLT2 inhibits CD4^+^ T cell metabolism, including glutamine replenishment, glycolysis, and oxidative phosphorylation (OXPHOS), thereby ameliorating renal injury.

**Figure 2:**
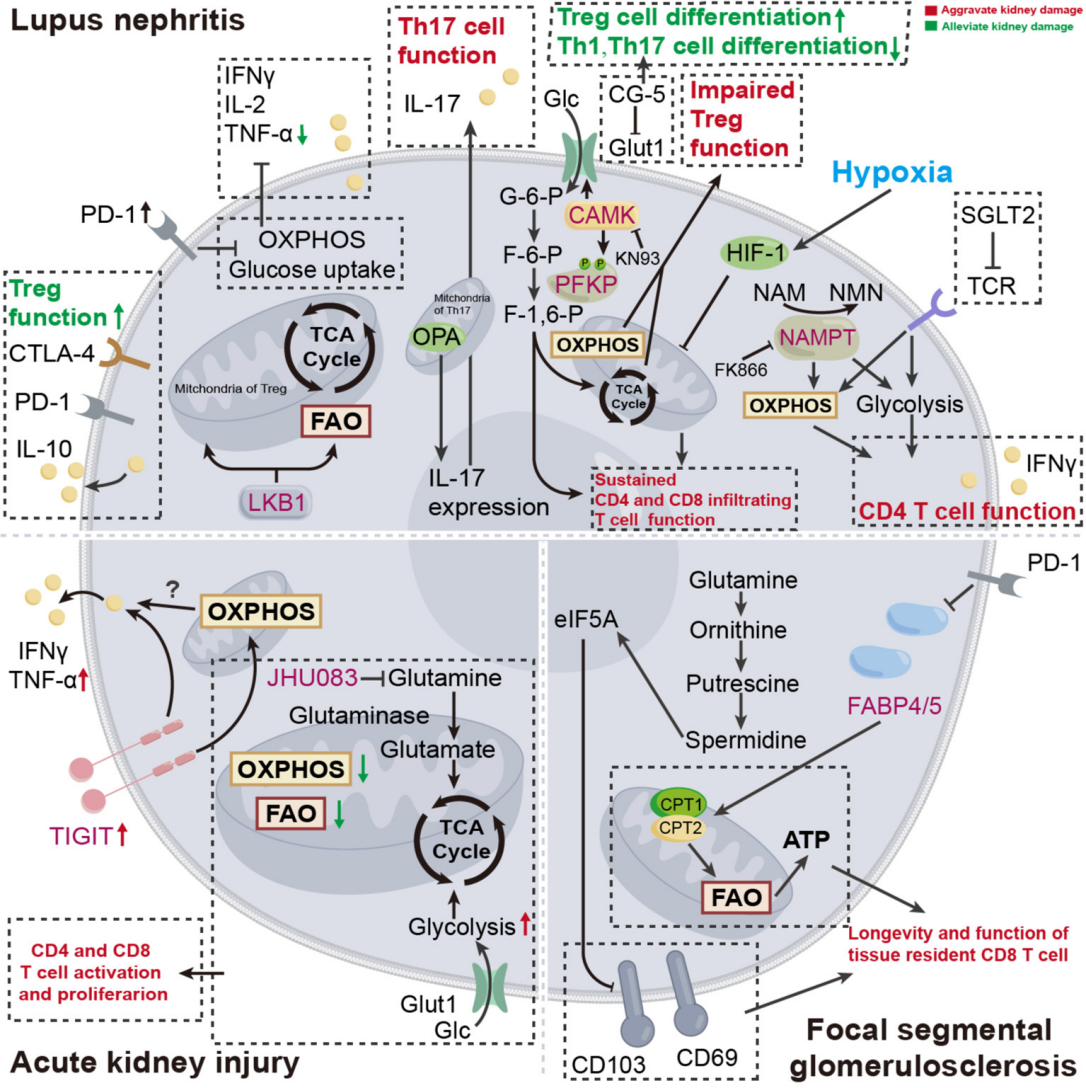
Disturbed cellular metabolism of T cell: pathogenesis of certain kidney disease. In the progression of certain kidney diseases, T cell metabolism undergoes a series of changes to provide necessary energy support. Targeting T cell metabolism is expected to offer new therapeutic options for kidney disease treatment. OXPHOS, oxidative phosphorylation; FAO, fatty acid oxidation; F-6-P, fructose-6-phosphate; F-1,6-P, glucose-6-phosphate; G-6-P, glucose-1-6-bisphosphate; Glc, glucose; GLUT, glucose transporters; OPA1, optic atrophy 1; LKB1, liver kinase B1; CAMK, calcium/calmodulin-dependent protein kinase; PFKP, 6-phosphofructokinase, platelet type; NAMPT, nicotinamide phosphoribosyltransferase; NMN, nicotinamide mononucleotide; NAM, nicotinamide; TIGIT, T-cell immunoreceptor with Ig and immunoreceptor tyrosine-based inhibitory motif domain; eIF5A, translation elongation factor eukaryotic translation initiation factor 5A; FABP, fatty-acid-binding proteins; SGLT2, sodium-glucose co-transporter 2.

Albeit the inspiring evidence on the metabolic modulation of CD4^+^ T cell subset in the kidney, however, the specific benefits to the kidney remain to be elucidated (**Figure 2**).

### 4.2 Metabolic modulation of CD8^+^ T cell subsets in kidney

Etiologically, the inflamed kidney tissue in certain contexts of kidney diseases is partly attributed to CD8^+^ T cell-derived pro-inflammatory cytokines. Given the functional discrepancy among various T cell subsets, the CD8^+^ T cell subsets that directly mediate kidney tissue damage are CTL. In lupus nephritis, the kidney infiltrating T cells is composed of an expanded cytotoxic population (123). However, the renal parenchymal tissues possess the capacity to sense inflammatory signals from infiltrating T cells and subsequently respond as increased expression of PD-L1. In the context of PD-1/PD-L1 signaling, partial renal-infiltrating CD4^+^ and CD8^+^ T cells exhibit exhausted phenotype, coupled with impaired glucose uptake and mitochondrial dysfunction, which may be responsible for limited cytokine production (124). This is considered a homeostatic mechanism for renal immune stability. Nevertheless, the proactive regulatory mechanism is insufficient to fully compensate for the infiltrating T cell-mediated kidney injury. As aforementioned, the infiltrating CD8^+^ T cells without high expression of PD-1, adapt to the hypoxic environment in the kidney by upregulating HIF-1. Subsequently, the upregulated HIF-1 alters the cellular energy demand towards glycolysis and halts its apoptosis, thus leading to kidney damage (115). However, further study, which focused on tumor-infiltrating CD8^+^ T cells, revealed that the exhausted phenotype is caused by both hypoxia and continuous antigen stimulation. To the authors' surprise, the hypoxia-induced exhaustion is not regulated by HIF-1α, but induced by dysfunctional mitochondrial products (125). Seemingly, this conclusion contradicts the data observed in lupus. Therefore, we tentatively hypothesize that the divergence in conclusions may be attributed to differences in hypoxic environments and antigenic stimulation.

Generally, differentiating between adaptive immune responses and innate immune responses on a temporal dimension, it is commonly believed that the early stages of immune response are predominantly characterized by innate immune responses. However, in the context of AKI, the concept that T cells respond at the early point is firmly rooted, and the prompt response is coupled with dramatic metabolic rewiring. Specifically, the T cell subsets (both CD4^+^ and CD8^+^) within the kidney exhibit diminished capacities in OXPHOS and FAO, while preserving glycolysis. The utilization of JHU083, a glutamine antagonist, reversed the T cell metabolic alterations induced by AKI and simultaneously inhibited the differentiation of T cells, thus alleviating kidney damage (**Table 1**) (126). However, given the global effect of glutamine antagonists, the off-target effect beyond T cells cannot be discounted (127). Briefly, JHU083 inhibits glutamine metabolism in CD4^+^ and CD8^+^ T cells, thus alleviating AKI.

The property of immunological memory within adaptive immune systems endows organisms with a rapid response when future immune scenarios emerge. Specifically, partial CD8^+^ T cells differentiate into CTL that secret cytokines, including granzyme B, TNFα, and IFNγ, to exert cytotoxic effects, while the remaining acquires longevity, as persisting in the form of memory T cell (128). Based on surface marker and migration pattern, memory T cells can be divided into three subsets: central memory T cells, effector memory T cells, and noncirculating tissue-resident memory T (T_RM_) cells. Given the tissue heterogeneity, the kidney T_RM_ cells are not entirely composed of CD8^+^ T cell subsets but also possess CD4^+^ T cell subsets. CD4^+^ T cell subsets govern the T_RM_ population in human kidney with simultaneous expression of CD69, CD103, and CXCR3 (129–132), and CD8^+^ T_RM_ cells in human kidney express CD69 and CD103 (133), while only expression of CD69 is found in mouse kidney (134). The expression of CD69 orchestrates T_RM_ cell maintenance in kidney (135), thus establishing itself as a potential therapeutic target. A recent study highlights that the generation of CD8^+^ T_RM_ is modulated by the glutamine/polyamine/hypusine axis. Mechanistically, glutamine-derived ornithine sequentially converts into putrescine and spermidine, the latter of which in turn facilitates the levels of translation elongation factor eukaryotic translation initiation factor 5A (eIF5A), thus dampening the expression of CD69. As a consequence, the generation of CD8^+^ T_RM_ is curbed (136).

Within the pathogenesis of kidney disease, different T_RMs_ fulfill its function. The data from human kidney specimens revealed that CD4^+^ T_RM_ cells significantly correlate with better kidney function and CD8^+^ T_RM_ significantly correlate with age (133), indicating the balance between CD4^+^ and CD8^+^ T_RM_ cells mediates the kidney immune homeostasis. A recent study revealed that, in the early-stage of AKI, the tissue-resident IL-33R^+^ and IL-2Ra^+^ Treg cells are prone to increase, thus alleviating renal interstitial fibrosis (137). However, the increasing abundance of CD4^+^ T_RM_ cells in the kidney, incited by infection, exhibit the Th17 signature of secreting IL-17, which subsequently exacerbates glomerular injury in the context of antineutrophil cytoplasmic antibody (ANCA)-dependent glomerulonephritis (GN) (129).

Tissue-resident T cells possess a distinct heterogeneity, not only for their surface marker but also the capacity of proliferation, constrained by tissue autonomous (138). Typically, the activation of naïve T cells is restricted by dual signal, among which the cognate antigen-MHC peptide complexes on the surface of APCs serve as signal 1, while costimulatory molecules act as signal 2. Therefore, to keep the maintenance within the kidney, the CD8^+^ T_RM_ cell necessitates cognate antigen and IL-15 in the context of transplantation or inflammation (139, 140). While, the kidney T_RM_ cells, under physiological conditions, are constantly maintained in a state of readiness for combat, for the reason that renal tubular epithelial cells are furnished with the capacity of stimulating T cells in an MHC-independent manner (138).

Bowman's capsule protects podocytes from the insulting incited by CD8^+^ T_RM_ cells, but the possibility of some unexpected outcomes, mostly accompanied by pathological context. cannot be excluded (141). For instance, in mice and patients with glomerular diseases, the increasing proportion of CD8^+^ T_RM_ cells potentiate the glomerular damage, and the administration of sparsentan, the dual angiotensin II (Ang II) receptor, and endothelin type A receptor antagonist, could partly alleviate the glomerular damage via down-regulating IL-15 (140).

Mostly, CD8^+^ T_RM_ is fueled by lipids for long-term residence. Inhibiting fatty-acid-binding proteins 4 and 5 (FABP4 and FABP5), the meditator for lipid uptake, impedes the maintenance, longevity, and function of CD8^+^ T_RM_ (142). Likewise, the CD8^+^ T_RM_ in gastric adenocarcinoma relies on fatty acid for cell survival, and the utilization of PD-L1 blockade diminished the expression of FABP4 and FABP5, which is considered an alternative target for checkpoint inhibitor in cancer treatment (143). Furthermore, Feng and his colleagues elucidate that fatty acid epigenetically improves the longevity and function of CD8^+^ T_RM_ by upregulating the transcription factor SCML4 (144).

JAK/STAT pathway can impact CD8^+^ T_RM_ cell metabolism, thereby orchestrating its differentiation and function (145). In lupus nephritis, JAK/STAT signaling mediates the proliferation and function of CD8^+^CD103^+^ T_RM_ cells, thereby resulting in kidney damage. Further, inhibiting JAK/STAT signaling by tofacitinib impaired the survival of Trm cells and restored kidney damage (146). These data suggest that the JAK/STAT pathway may mediate the fate of T_RM_ by modulating cellular metabolism. However, there is a lack of direct corroborative evidence, warranting further studies (**Figure 2**).

### 4.3 T cell metabolism in AKI vs CKD: a comparative perspective

AKI, manifested as an abrupt loss of excretory kidney function, can be caused by multiple insults, including ischemia sepsis, and nephrotoxic drugs. In this process, T cell act as a prominent pathogenic element, worsening the pre-existing kidney damage (147). Compared to traditional concept of adaptive immunity as tardive immune response, a considerable amount of research indicates that T cell infiltration occurs in the early stages of AKI and persists for a significant duration (148, 149). In addition, the development and progression of CKD are accompanied by a series of changes in innate and adaptive immune responses, with T cells serving as a key component of the adaptive immune system (150). Especially, chronic immune cell overactivation is the culprit in the development of CKD secondary to hypertension and diabetes. AKI and CKD exhibit differences in T cell subsets. To thoroughly explore T cell heterogeneity between AKI and CKD, a study adopted 2 established murine models of kidney regeneration and fibrosis, founding that Tregs preferentially accumulate in fibrotic mouse kidneys to limit initial inflammation and cellular injury (137). By employing high-resolution spatial information, the T cell subsets within kidney are well-characterized. In AKI mice, Tregs exhibit early immune aggregation within the kidney (151). The data from human kidney samples indicate a marked increase in the infiltration of various T cell subsets during the progression of CKD (152).

CKD is often associated with diabetes and hypertension, and chronic inflammation and T cell dysfunction are mutually causal in the progression of CKD. In the type 1 diabetic mice, infiltrating CD4^+^ T cells are increased in kidney, accompanied by excessive renal inflammatory factors (153). By using glycolysis inhibitors (FK15, inhibitor for PFKFB3), T cells exhibit metabolic exhaustion, leading to a delay in diabetes onset (154). Another intriguing study found that, compared to T2D, in prediabetic-state, Tregs regulate OXPHOS in effector T cells, leading to increased pro-inflammatory cytokine release, and the upregulation of the CD36 on Tregs may be the key culprit (155). Additionally, T cell senescence leads to increased secretion of inflammatory cytokines, accompanied by metabolic profile alterations (156). A large retrospective cohort study involving 523 T2D patients found a significant increase in CD8^+^ T cell senescence (CD28^-^, CD127^-^, and CD57^+^) with CKD progression (157). Transplantation of senescent T cells resulted in more severe inflammation and fibrosis in angiotensin-stimulated mice (16). The hypoxic state of AKI enhances T cell glycolysis, leading to increased proliferation, which can be reversed by glutamine inhibitors (126). The exploration of how CKD and AKI microenvironments influence T cells remains in its early stages, with significant gaps in knowledge yet to be filled.

## 5 Conclusion

The intricate interplay between immune homeostasis and metabolism is fundamental to the pathophysiology of T cells. In this review, we highlight T cell metabolism as a potential therapeutic target for kidney disease and review recent advancements in T cell immunometabolism and the renal microenvironment in the disease context.

Despite many distinguished studies that emerged in the field of T cell metabolism, the majority focused on anti-tumor. There is limited direct evidence for the role of T cell metabolism in the kidneys. Moreover, the investigation of related mechanisms remains in its early stages, with few in-depth mechanistic analyses available. Many questions remained unsolved in the part about how the cellular metabolism within T cells regulates its activation, differentiation, effector function, and exhaustion, thus potentiating kidney damage. Hence, to fill the gaps in this field and better translate basic science into clinical practice, more high-quality research is needed.
